# Molecular diagnosis of cutaneous leishmaniasis and identification of the causative *Leishmania* species in Morocco by using three PCR-based assays

**DOI:** 10.1186/1756-3305-7-420

**Published:** 2014-09-04

**Authors:** Tarik Mouttaki, Manuel Morales-Yuste, Gema Merino-Espinosa, Soumiya Chiheb, Hassan Fellah, Joaquina Martin-Sanchez, Myriam Riyad

**Affiliations:** Faculty of Medicine and Pharmacy, Centre for Doctoral Studies in Health Sciences, Casablanca, Morocco; Department of Parasitology, Faculty of Pharmacy, Granada, Spain; Department of Dermatology, University Hospital Ibn Rochd, Casablanca, Morocco; Laboratory of Parasitology-Mycology, Faculty of Medicine and Pharmacy, Casablanca, Morocco; Research Team on Cutaneous Leishmaniasis, Faculty of Medicine and Pharmacy, 19 rue Tarik Ibn Ziad, BP. 9154 Casablanca, Morocco

**Keywords:** Cutaneous leishmaniasis, *L. tropica*, *L. major*, *L. infantum*, Direct examination, Culture, PCR, PCR-RFLP, 13A/13B primer, Lmj4/Uni21 primer, LITSR/L5.8S primer, Morocco

## Abstract

**Background:**

The diagnosis of cutaneous leishmaniasis (CL) might be difficult, in particular in endemic areas where different species of *Leishmania* can cause lesions of very similar appearance and where other skin diseases with similar clinical symptoms occur. Even today, the parasitological diagnosis of CL remains the gold standard and it is based on the direct identification of amastigotes in microscopy smears and/or culture of promastigotes from infected tissues. Although these techniques are highly specific, they are not sensitive enough. The objective of this study is to contribute to improving the diagnosis of CL and the identification of *Leishmania* species in Morocco by comparing three PCR-based assays applied directly on dermal samples.

**Methods:**

A total of 58 patients presenting with cutaneous lesions suggestive of CL were sampled for parasitological diagnosis by direct examination (DE), culture in NNN medium, two kinetoplast DNA (kDNA) PCRs (Lmj4/Uni21 and 13A/13B primers) and one rRNA gene internal transcribed spacer 1 (ITS1) PCR (LITSR/L5.8S primers). The techniques were statistically analyzed and compared.

**Results:**

According to our consensus positive, 44 out of 58 samples were true positives. The 13A/13B-PCR and ITS1-PCR showed the highest sensitivities (100%). Parasite microscopy and culture detected 43% and 29% of the true positives, respectively, while culture and microscopy together improved sensitivity to 52%. PCRs 13A/13B and ITS1 were associated to four and one false positives, respectively, while the other assays were 100% specific. Furthermore, the ITS1-PCR-RFLP assay clearly identified the *Leishmania* species for all the true positives (44/44), whereas Lmj4/Uni21-PCR identified 35/44 samples. The comparison between the *Leishmania* molecular characterizations and the expected species according to the national data from the Ministry of Health indicate 7 discrepant results.

**Conclusions:**

The PCR-based assays tested on our samples increased the speed and sensitivity of the diagnosis of CL compared to the conventional techniques. Furthermore, we showed that we can not base the species identification on the national data from the Ministry of Health. Finally, we suggest the use of PCR-ITS1-RFLP for diagnosis and simultaneous identification of the species in the Moroccan epidemiological context, but also in similar areas of the Mediterranean Basin.

## Background

Leishmaniases are vector-borne parasitic diseases caused by protozoa of the genus *Leishmania* (Kinetoplastida: Trypanosomatidae) [1]. The clinical spectrum of these diseases range from self-resolving cutaneous lesions to visceral forms, fatal if left untreated.

Leishmaniases remain one of the world’s most devastating neglected tropical diseases.

According to the World Health Organization (WHO), up to 350 million people are at risk in 98 countries around the world. It is considered that approximately 12 million people are currently infected, and 2 million new infections occur every year, of which an estimated 1.5 million cases are cutaneous [2, 3]. Cutaneous leishmaniasis (CL) occurs either as zoonotic or anthroponotic infections, commonly caused by various species of *Leishmania*, namely *Leishmania major*, *L. tropica*, *L. infantum*, *L. aethiopica*, *L. mexicana*, *L. amazonensis*, or *L. braziliensis* [4] *.* They cause skin ulcers on the exposed parts of the body and scars can cause serious social prejudice [5].

The diagnosis of CL can be difficult, especially in endemic areas where different species of *Leishmania* can cause lesions of very similar appearance and where other skin diseases with similar clinical symptoms occur. This may impair or prevent the proper course of treatment: for instance CL caused by viscerotropic species (e.g. *L. infantum*) may need an appropriate therapeutic monitoring [6].

Presumptive diagnosis of CL is based on clinical symptoms. Even today, the parasitological diagnosis remains the gold standard and it is based on the direct identification of amastigotes in microscopy smears and/or culture of promastigotes from infected tissues [7]. Although these techniques are highly specific, they are not sensitive enough and are generally time-consuming [8]. In addition, culture should be kept for at least one month before definitive negative result [9]. When promastigotes are isolated, additional techniques must be used for species characterization: multilocus enzyme electrophoresis analysis remains the current gold standard, but it requires mass culturing of the parasites [10].

In Morocco, CL still remains a major public health problem caused by three *Leishmania* species. *L. major* is the causative agent of zoonotic CL characterized by successive endemic and epidemic cycles in arid presaharian areas of the country [11]. *L. tropica* is the causative agent of anthroponotic CL which is hypoendemic in rural areas of central Morocco and expanded to northern areas in the 1990s; currently it is not only characterized by the widest geographical distribution in Morocco but also in North Africa with the highest incidence rate [12, 13]. Finally, the viscerotropic species *L. infantum* is also involved in sporadic CL in the northern areas of VL distribution; the first case was documented in 1996 in a Northern active focus of Canine leishmaniasis in the Rif Mountains and recently a new *L. infantum* CL focus (Sidi Kacem) was also identified in the North [13–15].

Even today, the diagnosis of CL in Morocco is mainly based on clinical symptoms and microscopic examination of smears; culture is carried out only in some specialized laboratories. Furthermore, the national data on the geographical distribution of *Leishmania* species together with the clinical presentation of the resulting disease are frequently used for species identification. However, the increased clinical diversity and the geographical extension of *L. tropica* beyond the areas where it was previously recorded, make these criteria inadequate for species identification [16]. Thus, the diversity of the species associated with human CL and the changing epidemiological situation in Morocco highlights the need for the development of a method allowing the diagnosis and the characterization of the infective species: this method has to be preferably simple, cheap, and less time consuming than the gold standard. In this frame, PCR-based methods offer an alternative approach to traditional techniques [17]. A crude search in the Pub-Med database revealed that since 1989, more than 700 articles on PCR diagnosis of leishmaniases have been published, in which a multitude of gene targets, protocols, and applications are described, including genus and/or species-specific PCR, ranging from low-tech to high-tech approaches [7]. The PCR-based assays currently constitute the main molecular diagnostic approach for the detection and identification of *Leishmania* parasites in clinical samples [7]. PCR primers targeting the kinetoplast and ribosomal DNA genes are amongst the most commonly used for the diagnosis and/or identification of *Leishmania* species in the Old World and they provide good results [17–22]. For our purpose and according to the literature, the 13A/13B and Lmj4/Uni21 primers derived from kDNA sequences, and LITSR/L5.8S primers targeting the internal transcribed spacer-1 of the rDNA appeared to be suitable in our epidemiological context.

Thus, given the diversity of the clinical presentations and the circulation of three potentially responsible species of CL in Morocco, the objective of this study is to contribute to improving the diagnosis of the disease and the identification of the causative *Leishmania* species in Morocco by comparing three selected PCR-based assays applied directly on dermal samples.

## Methods

### Ethics statement

This study was conducted according to the principles specified in the Declaration of Helsinki and under the local ethical guidelines (Comité d’Ethique pour la Recherche Biomédicale, Faculty of Medicine and Pharmacy, Casablanca, Morocco).

### Patients and samples

Amongst the patients consulting at the Department of Dermatology - Ibn Rochd Hospital, Casablanca -, 58 presented with cutaneous lesions suggestive of CL; they were sampled by the dermatologist for parasitological and molecular diagnosis.

Out of these 58 patients sampled, 23 were men and 35 were women with an average age of 33 ± 24 years: patients were mostly younger than 12 years and older than 49 years. The duration of lesion prior to sampling ranged from 2 weeks to 48 months (Table 1). Furthermore, fifty two patients (52/58) either lived in an endemic area of CL or had a history of travel to a known CL focus, according to the Ministry of Health data based on cases reported by the clinicians and epidemiological case detection studies.

**Table 1:** **Time evolution of skin lesions prior to sampling**

Duration (months)	Number of patients
**<2**	11
**2-4**	9
**4-8**	26
**>8**	12
**Total**	58

The dermal syringe-sucked fluid was collected under sterile conditions from the border of active skin lesions from each patient as follows: the lesions were cleaned with alcohol, and 0.1 to 0.2 ml of sterile saline solution was injected using a syringe (1-ml, 25-gauge needle) into the nodule and the needle was rotated gently several times. A small amount of saline solution was injected into the tissue, and then aspirated. Three syringes were used for each patient in order to take samples of the dermal fluid for culture, PCR assays, and for microscopy examination. The sample meant for the PCR was conserved at -30°C until processing.

An information sheet specifying the age, the sex, and the place of infection was filled, in order to compare the species characterization results with the data of the Ministry of Health.

### *Leishmania*strains

Reference strains of *L. major* (MHOM/IL/81/Friedlin), *L. tropica* (MHOM/SU/74/K27) and *L. infantum* (MHOM/ES/90/LEM2205) were included: frozen strains at -80°C were thawed and then cultured in RPMI 1640 medium in order to prepare the DNA from the references (See corresponding paragraphs).

### Direct examination (DE)

Syringe-sucked dermal fluid was smeared onto glass slides, air dried and fixed with absolute methanol, allowed to dry, and then stained with Giemsa. The whole slide was analyzed with a 100× immersion objective. All the slides were inspected for the presence of amastigote forms at least twice before delivering the final result.

### Parasite culture

The syringe-sucked dermal fluid was inoculated in sterile conditions to NNN medium supplemented with 1% sterile human urine [23], and then incubated at 24-28°C. The supernatant was examined for parasite growth by light microscopy every three days and subcultured once a week for 6 weeks before they were reported as negative. Positive cultures were transferred to RPMI-1640 supplemented with 10% fetal calf serum for mass culturing.

The reference strains were also cultured as described above after thawing.

### DNA isolation

DNA was extracted from each clinical sample (100 μl volume) preserved at -30°C and from the reference strains culture (volumes corresponding to 1.10^6^ parasites) using the Pure Link™ Genomic DNA Mini Kit (Invitrogen, UK) according to the manufacturer’s instructions. The DNA was kept at -20°C until PCR processing.

### *Leishmania*serial dilution assay

The lowest detection threshold of each PCR method was estimated using a serial of dilutions of DNA extracted from 7-day-old promastigotes cultures of two reference strains of *L. major* and *L. tropica,* which were counted using a Thoma haemocytometer. They were resuspended in 200 μl of whole human blood adjusted to 1000 parasites and the DNA was extracted. From this DNA, serial dilutions were made in order to obtain DNA yield equivalent to 100, 50, 10, 5, 1, 0.5, 0.1, and 0.01 parasites per PCR tube. The test was repeated twice.

### PCR amplification and internal control

The three PCR assays tested are described hereafter (Table 2):

- The Minicircle kDNA was amplified using two different sequence pairs of primers: 13A/13B and Lmj4/Uni21 as described by Reale et al. [24] and Anders et al. [25] respectively.
- ITS1 PCR assay was carried out as described by Schönian et al. [26], using the primers LITSR/L5.8S to amplify the ribosomal ITS1 region.

**Table 2:** **Primer sequences and main conditions of the PCR methods used**

			PCR conditions				
Primer name	Primer sequence	Amplification conditions	MgCl _2_ (mM)	dNTP (mM)	Primer (μM)	Unit of Taq DNA Polymerase	DNA (μL)
**13A**	5′-GTG GGG GAG GGG CGT TCT-3′	94°C for 4 min, 94°C for 1 min, 60°C for 1 min, 72°C for 1 min, 72°C for 10 min (30 cycles)	1.5	0.2	1	1	2.5
**13B**	5′-ATT TTC CAC CAA CCC CCA GTT-3′						
**Lmj4**	5′-CTA GTT TCC C GC CTC CGA G-3′	94°C for 4 min, 94°C for 1 min, 55°C for 1 min, 72°C for 1 min, 72°C for 10 min (35 cycles)	1.5	0.2	1	1	2.5
**Uni21**	5′-GGG GTT GGT GTA AAA TAG GCC-3′						
**LITSR**	5′-CTG GAT CAT TTT CCG ATG-3′	94°C for 4 min, 94°C for 40 sec,53°C for 30 sec, 72°C for 1 min, 72°C for 10 min (40 cycles)	2	0.2	0.5	1.5	10
**L5.8S**	5′-TGA TAC CAC TTA TCG CAC TT 3′						
**GH20**	5′-GAA GAG CCA AGG ACA GGT AC-3′	94°C for 4 min, 94°C for 30 sec, 54.5°C for 1 min, 72°C for 1.3 min, 72°C for 10 min (40 cycles)	2.5	0.2	0.4	1	2
**PCO4**	5′-CAA CTT CAT CCA CGT TCA CC-3′						

The PCR conditions were optimized for each assay by using the DNA from the three reference strains of *Leishmania*. All PCRs were carried out in a 25 μl volume except for the ITS1 PCR (in 50 μl), and performed in a DNA thermal cycler (Techne Genius Thermal Cycler, USA).

To avoid DNA contamination, the different steps of the technical procedures were carried out in separate areas with dedicated pipette batches and decontamination procedures according to standard recommendations [27]. The presence of possible PCR reaction inhibitors was ascertained by testing the samples with negative PCR results for *Leishmania* using the specific primers for the human beta-globin gene GH20/PCO4 as described by Saiki et al. [28].

A PCR result was considered positive when a band of the expected size was obtained: ~120 bp for 13A/13B (*Leishmania* genus), ~650-800 bp for Lmj4/Uni21 (~650 bp for *L. major* and ~800 bp for *L. tropica/ L. infantum*), ~300-350 bp for ITS1 (*Leishmania* genus), and ~268 bp for β-globin gene PCR.

After the amplification, PCR products were analyzed by 1–2.5% agarose gel electrophoresis/ethidium bromide staining and visualized under UV transilluminator.

### RFLP analysis

The ITS1 PCR product was digested with HaeIII enzyme (Invitrogen, UK) according to the manufacturer’s instructions. The restriction fragments were analyzed by 2.5% agarose electrophoresis/ethidium bromide staining: they were visualized by UV light. The digestion of ITS1 products reveals three bands for *L. infantum* (200, 100, and 50 bp), two bands for *L. tropica* (220 and 50 bp), and two for *L. major* (220 and 127 bp).

### Statistical analysis

By consensus we considered specimens as true positives when cultures or stained tissue smears or at least two PCR assays were positive for *Leishmania* DNA. Sensitivity, specificity, positive predictive values (PPV), negative predictive values (NPV), and Cohen’s kappa coefficient (χ) were determined. Cohen’s kappa coefficient is a measure of the agreement between two tests beyond that expected by chance, where 0 is chance agreement and 1 is perfect agreement [29, 30]. The strength of agreement is defined as follows: poor (<0.20), fair (0.21-0.40), moderate (0.41-0.60), good (0.61-0.80), and very good (0.81-1.00).

The association between different variables was estimated using a binary logistic regression analysis (SPSS software v 20.0), at a significance level of 5%.

## Results

Of the 58 specimens collected from the patients with suspected CL, and according to our consensus criteria, 44 samples were true positives with a female predominance (OR = 3.857; IC 95%: 1.090-13.652; p = 0.036) and 14 were true negatives. CL diagnosis was negatively associated with the duration of lesions prior to sampling (Odds Ratio = 0.923; Confidence Interval 95%: 0.858-0.993; p = 0.033). The results obtained with the different assays are presented in Tables 3 and 4.

**Table 3:** **Results of the different diagnostic methods**

Direct examination	Culture	13A/13B PCR	Lmj4/uni21 PCR	ITS1 PCR	Final interpretation*	Number (total: 58)
-	-	+	+	+	+	16
-	-	+	-	+	+	5
-	-	-	-	+	-	1
-	-	+	-	-	-	4
-	-	-	-	-	-	9
-	+	+	+	+	+	4
+	+	+	+	+	+	9
+	-	+	+	+	+	6
+	-	+	-	+	+	4

*According to the consensus positive.

**Table 4:** **Results and performance of the five diagnostic assays for cutaneous leishmaniasis**

Diagnostic method	Number of positives	True positives	Number of negatives	True negatives	Sensitivity (%)	Specificity (%)	PPV (%)	NPV (%)	χ ± [SE]
**Direct examination**	19	19	39	14	43	100	100	36	0.270 ± 0.110
**Culture**	13	13	45	14	29	100	100	31	0.170 ± 0.102
**13A/13B PCR**	48	44	10	10	100	71	92	100	0.791 ± 0.101
**Lmj4/Uni21 PCR**	35	35	23	14	79	100	100	61	0.652 ± 0.106
**ITS1-PCR**	45	44	13	13	100	93	98	100	0.950 ± 0.040
**PCR c**	44	44	14	14	100	100	100	100	
**Total**		**44**		**14**					

**PPV**: Positive Predictive Value; **NPV:** Negative Predictive Value; **PCRc**: PCR consensus results.

95% CI for sensitivity, specificity, PPV and NPV.

### Stained tissue smears and parasite culture

Both parasite cultures and DE were highly specific (100%) for the diagnosis of CL, and when considered together they correctly identified 23/44 of the true positive specimens, while 14 of these true positives (14/23) were detected only by one of the two methods. Their respective sensitivities (microscopy: 43%; culture: 29%) were improved when both methods were combined (52%). The level of agreement, Cohen’s kappa coefficient (χ ± standard error [SE]), between culture and DE (0.330 ± 0.150) was fair. Furthermore, the levels of agreement between positive cultures or DE and the consensus results (true positive and true negative samples) were respectively 0.170 ± 0.102 (poor) and 0.270 ± 0.110 (fair) - Table 4 - . On the other hand all the 23 specimens positive by culture and/or DE were confirmed by at least two of the PCR assays (Table 3). The DE and culture results have a negative correlation with the time evolution of skin lesions (OR = 0.861; CI 95%: 0.740-1.001; p = 0. 05).

### *Leishmania*DNA serial dilution assay

Serial dilutions of DNA of the reference parasite strains led to different detection thresholds of each PCR method: 0.1 parasite cells per PCR tube (p/t) for 13A/13B primers, 1 p/t for LITSR/L5.8S primers, and 5 p/t with Lmj4/Uni21 primers. The results were reproducible.

### Comparison of the PCR assays for CL diagnosis

Amongst the 58 samples, 48 were positive by the 13A/13B PCR, of which 44 were true positives (Table 4): the sensitivity and specificity values are 100% and 71% respectively; the PPV and NPV for this assay were 92% and 100%, respectively. The level of agreement between the consensus results and the 13A/13B PCR results was good: 0.791 ± 0.101.

The ITS1 PCR displayed a high sensitivity (100%), diagnosing 44/44 of the true positives (Table 4). The PPV and the NPV for the ITS1 assay were 98% and 100% respectively. The level of agreement between the consensus results and the ITS1 PCR was very good: 0.950 ± 0.040.

The sensitivity of the Lmj4/Uni21 PCR was 79% while its specificity and the PPV were 100%, since no false positives were found (Table 4). The NPV of this assay was 61%. The measure of agreement between the Lmj4/Uni21 PCR and the consensus results was also good: 0.652 ± 0.106. The results of the PCR consensus (PCR consensus were considered positive when at least two PCR assays were positive) had a negative correlation with the time evolution of skin lesions (p = 0. 03).

### Identification of *Leishmania*species

**Figure 1:**
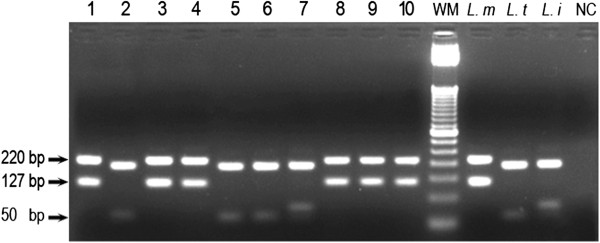
**2.5% Agarose gel electrophoresis of ITS1-PCR restriction fragments.** Wells: 1 – 10: patient samples; *L. m*: reference DNA of *L. major*; *L. t*: reference DNA of *L. tropica*; *L. i*: reference DNA of *L. infantum*; NC: negative control sample (H2O); WM: Weight Marker (50 bp DNA Ladder; Invitrogen Life Technologies, Brazil).

**Figure 2:**
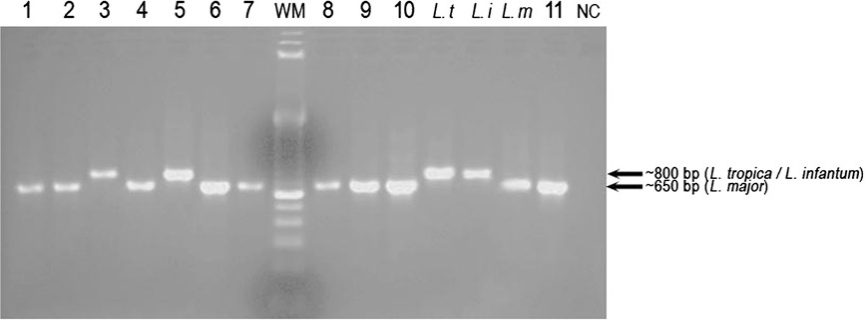
**2.5% Agarose gel electrophoresis of Lmj4/Uni21-PCR products.** Wells: 1 – 11: patient samples; *L. t*: reference DNA of *L. tropica*; *L. i*: reference DNA of *L. infantum*; *L. m*: reference DNA of *L. major*; NC: negative control sample (H2O); WM: Weight Marker (100 bp DNA Ladder; Invitrogen Life Technologies, Brazil).

*Leishmania* species identification was carried out using the ITS1 and Lmj4/Uni21 PCRs. The ITS1 PCR-RFLP assay clearly identified the species for all the true positives (44/44) (Figure 1, Table 5), whereas Lmj4/Uni21 PCR identified 35/44 samples; this latter primer did not allow the differentiation between *L. tropica* and *L. infantum* (Figure 2, Table 5).

**Table 5:** ***Leishmania*** **species identification**

PCR assays	***L. major***	***L. tropica***	Total
**ITS1 PCR**	19	25	44
		***L. tropica/L. infantum***	
**Lmj4/Uni21 PCR**	15	20	35

NB: one clinical sample identified as *L. infantum* by ITS1 PCR but negative by the other techniques is not included in this table.

No species discrepant results were noted between the two techniques, however, an additional sample, not considered as a true positive according to our consensus criteria was characterized as *L. infantum* by the ITS1 PCR (Figure 1).

On the other hand, 7 discrepant results were obtained between the consensus *Leishmania* diagnosis and the national data from the Ministry of Health (Table 6).

**Table 6:** **Species characterization vs. known endemic focus**

		Origin of infection (known foci of)*						Total of true positives
		***L. tropica***	***L. major***	***L. tropica***and ***L. major***	***L. tropica***and/or ***L. infantum***	Risk area non identified	Free CL area	
**Species characterization**	*L. tropica*	13	2	0	7	2	1	25
	*L. major*	3	13	1	1	1	0	19
**Total**		16	15	1	8	3	1	44

*According to the national data from the ministry of health.

NB: one clinical sample identified as *L. infantum* by ITS1 PCR but negative by the other techniques is not included in this table.

### Detection of PCR inhibition

PCR inhibitors are a setback in clinical samples from different sources. Successful amplification of the beta-globin gene fragments (268 bp) indicates that the DNA sample is adequate for PCR analysis and that no PCR inhibitors are present.

In our case 49/58 of samples were detected by at least one PCR assay for *Leishmania* DNA (Table 3), it is supposed there were no PCR inhibitors in these samples. Nevertheless, for nine samples that were negative by the three PCR assays we detected inhibition of the PCR in only one sample when using the internal control: it might well be due to the presence of a PCR inhibitor that was not removed during the extraction method process, or the degradation of the target DNA, or more likely due to insufficient amount of biological material. Unfortunately we could not repeat the tests for the corresponding patient.

## Discussion

CL disease represents a major public health problem in Morocco: in 2011, more than 4,000 cases were reported [31]. According to the WHO, women and children are the most affected groups; this fact was consistent with our study where women were almost 4 times more affected with CL than men (p = 0.036). They may be more vulnerable because during the hot season they mostly sleep indoors at night, while men sleep outside the home. The *Phlebotomus* species in general and the female *Phlebotomus* in particular tended to be more endophilic than exophilic, although 40% of the *Phlebotomus* females are collected outside [32].

The classical methods have limitations, especially with regard to sensitivity ranging from 17 to 83% for DE [33–35], and from 27 to 85% for culture [18, 34, 35]. In our study, 14 out of 23 samples positive by DE and/or culture were detected by either of these methods (Table 3), leading to low sensitivity values (Table 4). This low performance of the parasitological diagnosis might be due to the parasite load, the sampling site (the more recent lesion is recommended in case of multiple lesions), or the evolution of skin lesions at the time of the clinical examination (the number of the parasites in skin lesions decreases in chronic CL). In our study, 38/58 samples (65%) were associated with long time evolution of skin lesion before sampling, older than 4 months (Table 1, Table 4). The responsible species is also involved especially for cultures: *L. infantum* from dermal lesions is reported to be more difficult to isolate [36]. Furthermore, the technical expertise of the biologist and the microbiological contamination of the culture media can also impact negatively on the sensitivity. For all these reasons the high specificity of DE and culture is of little relevance especially in endemic areas: indeed, a positive result with either of these classical methods should always be considered a true positive.

In our conditions, the 13A/13B primers showed higher efficiency compared to the other primers, being able to detect an equivalent of 0.1 promastigote cells per PCR tube. This may be explained in part by the higher copy number of the kDNA target (approximately 10,000 copies) which is 50 and 250 fold higher than ITS1 target regions (40 to 200 copies). Furthermore, the smaller size of the 13A/13B PCR products (120 bp) may be amplified more efficiently than larger fragments (320 bp for ITS-1, and 650–850 bp for Lmj4/Uni21 primers). Lachaud et al. [37] found a better value of 10^-4^ parasites per reaction with 13A/13B PCR but the *Leishmania* serial dilution assay was performed by seeding the promastigotes into the buffy coat [37]. Schönian et al. [26] showed that ITS1 PCR could detect leishmanial DNA equivalent to 0.2 parasites per PCR assay using filter paper spotted with peripheral blood mixed with culture promastigotes [26]. Anders et al. [25] reported that the Lmj4/Uni21 PCR could detect 0.25 parasites using pure DNA of promastigotes culture; however, they specified that these primers were often not sensitive enough to detect a single parasite from clinical samples [25]. Thus, the broad range of the reported detection rates can be explained by several parameters of which: the kind of the clinical material, the DNA extraction method that can affect the quality of DNA and the Taq polymerase used as well [25]. Furthermore, we showed that the results of the PCR assays are negatively correlated with the time evolution of the cutaneous lesions.

In the present study and in order to enhance the specificity of our PCR assays, the samples were considered molecular-positives when at least two PCR assays were positive: 21 CL patients missed by both microscopic examination and culture (21/35) resulted positive by at least 2 PCRs (Table 3). These samples may have contained very few parasites.

Both 13A/13B and ITS1 PCR assays gave sensitivities of 100% based on our consensus positive, and 13A/13B PCR identified four samples missed by the ITS1 PCR (Table 3). Bensoussan et al. [21] reported sensitivities of 13A/13B and ITS1 PCR assays of 98.7% and 91% respectively, while Azmi et al. [20] reported respective sensitivities of 92% and 63.5%. These different rates for the same primers can be explained by various factors such as the length of infection affecting the parasite loads, and also the different consensus standards considered in these studies.

Although the Lmj4/Uni21 primers amplifies a fragment of a highly repetitive kDNA region, this PCR showed the lowest sensitivity (Table 4); Anders et al. [25] also considered this method limited in terms of sensitivity (less than 10 parasites per tube). This can be attributed in part to the larger size of the amplification product (~650 - 800 bp), but also to the fact that Lmj4 primer was designed on the basis of a variable region of the minicircle sequence. Only one study conducted by Kumar et al. [22] reported that Lmj4/Uni21 PCR is more sensitive than the ITS-1 but this result is based on a smaller sample size (29 cases), and it may also be due to the biopsy material used instead of lesion aspiration [22].

Finally, in our conditions the kDNA 13A/13B PCR is a highly sensitive diagnostic assay and thus seems a valuable tool for the diagnosis of CL, however, it is not able to differentiate between the *Leishmania* species. The Lmj4/Uni21 and ITS1 PCRs have the additional advantage that the species can be distinguished. In our context the species identification is not only important because three species can be responsible for the disease, but also for other purposes: a targeted therapy, and the study of the epidemiology and dynamics of the disease. This latter point is important to design well targeted control strategies. In the Moroccan frame, the existence of three clinico-epidemiological forms of CL makes clinical and epidemiological criteria alone definitely inadequate for species designation. As shown in Table 6, six patient samples were characterized as *L. tropica* or *L. major* while these species were not reported by the Ministry of Health in the corresponding risk area, and one patient from a CL free area was confirmed as a positive CL case [31].

Concerning the ITS1 PCR, the restriction of the amplification products of the three reference strains gave three different patterns unambiguously differentiated into *L. tropica*, *L. infantum* or *L. major*. All our dermal samples ITS1 PCR positives displayed different restriction patterns associated with *L. tropica* (25/45) or *L. major* (19/45); one sample negative by the other PCR assays was identified as *L. infantum* (Table 5). Given that this parasite species has a viscerotropic potential, it requires special monitoring even if it is not a confirmed case by our consensus. Concerning the Uni21/Lmj4 PCR, this PCR method was not very sensitive and it could not discriminate between *L. tropica* and *L. infantum* infections. In Northern areas of Morocco where both species may coexist, this primer is of little interest.

## Conclusions

In conclusion, the PCR-based assays tested on our dermal aspirates increased the speed and sensitivity of species-specific leishmaniasis diagnosis compared to the conventional techniques. In the Moroccan epidemiological context, we showed that we cannot base the species identification either on the clinical aspects of the skin lesions or on the data from the Ministry of Health. We suggest the use of the ITS1 PCR-RFLP assay for diagnosis and simultaneous identification of the species. The 13A/13B PCR can be recommended in well-known foci and in usual clinical presentations. However, PCR-based protocols still need standardization and optimization; furthermore, the molecular approaches remain expensive and require technological expertise especially for remote areas where leishmaniasis is endemic. Thus, in the absence of PCR-based assays, we recommend that both microscopic examination and parasite culture should be employed together for CL diagnosis; negative samples should be subjected to PCR diagnosis whenever possible when they are associated with a suggestive clinical presentation, or the possibility of infection with a viscerotropic species, and when other dermal pathologies have been excluded.
